# Long-distance connections in the Copper Age: New evidence from the Alpine Iceman’s copper axe

**DOI:** 10.1371/journal.pone.0179263

**Published:** 2017-07-05

**Authors:** Gilberto Artioli, Ivana Angelini, Günther Kaufmann, Caterina Canovaro, Gregorio Dal Sasso, Igor Maria Villa

**Affiliations:** 1 Department of Geosciences, Università di Padova, Padova, Italy; 2 INSTM, Consorzio Interuniversitario Nazionale per la Scienza e Tecnologia dei Materiali, Firenze, Italy; 3 Department of Cultural Heritage, Università di Padova, Padova, Italy; 4 Museo Archeologico dell’Alto Adige/Südtiroler Archäologiemuseum, Bolzano/Bozen, Italy; 5 Centro Universitario Datazioni e Archeometria, Università di Milano Bicocca, Milano, Italy; 6 Institut für Geologie, Universität Bern, Bern, Switzerland; New York State Museum, UNITED STATES

## Abstract

25 years after the discovery in the Ötztal Italian Alps, the 5,300-year-old mummy keeps providing key information on human biological and medical conditions, aspects of everyday life and societal organization in the Copper Age. The hand axe found with the body of the Alpine Iceman is one of the rare copper objects that is firmly dated to the early Copper Age because of the radiocarbon dating of the axe wooden shaft. Here we report the measurement of the lead isotope ratios of the copper blade. The results unambiguously indicate that the source of the metal is the ore-rich area of Southern Tuscany, despite ample evidence that Alpine copper ore sources were known and exploited at the time. The experimental results are discussed within the framework of all the available coeval archaeometallurgical data in Central-Southern Europe: they show that the Alps were a neat cultural barrier separating distinct metal circuits. The direct evidence of raw metal or object movement between Central Italy and the Alps is surprising and provides a new perspective on long-distance relocation of goods and relationships between the early Copper Age cultures in the area. The result is in line with the recent investigations re-evaluating the timing and extent of copper production in Central Italy in the 4^th^ millennium BC.

## Introduction

**Fig 1:**
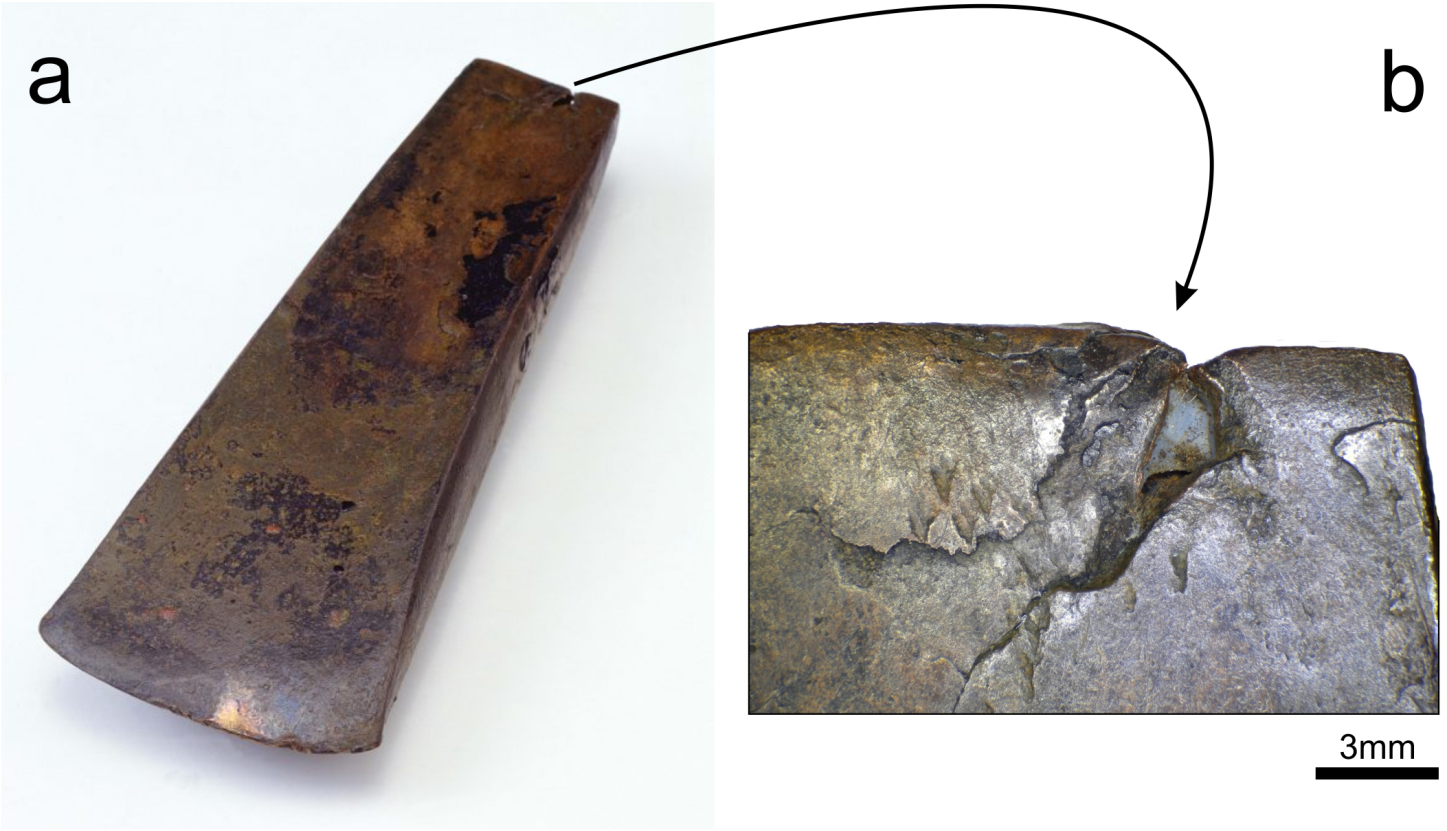
The Iceman hand axe. a) It is the oldest axe found complete of the copper blade, hide strips, birch tar, and handle made of yew wood, so that it has been carefully dated by radiocarbon methods (figure from www.iceman.it, modified) b) Casting defects and deformation in the talon of the copper blade. The microsample here analyzed was extracted from the major cavity.

The Tyrolean Iceman, a 5,300-year-old (Copper Age) natural mummy discovered in the Italian Ötztal Alps in 1991 [1–2], provides direct archaeological and anthropological perspectives on prehistoric Europe. Over two decades of scientific analyses on the mummy and related objects have provided unprecedented knowledge on ancestry, diet, tools, lifestyle, health and attire [3–9] of humans living in a relatively unexplored period of European prehistory. Understandably the research to date has been largely focused on the biological, medical, and forensic aspects, due to the exceptional preservation of the body and of his garments, and of course because of our need to develop social relationship with the mummified body [10] up to the point of making the frozen body a popular character, nicknamed Ötzi. Recently a number of scientific investigations finally addressed the archaeological issues related to the inorganic tools and implements found with the body, especially the copper blade (Fig 1), which is the oldest prehistoric metal blade found complete of the ropes and wooden handle in the world [11].

The first investigations on the Iceman's copper axe mostly involved typological aspects [12–13] and only very preliminary chemical assessments [14–15]. Then, about fifteen years ago, permission was granted to carry out detailed non-invasive crystallographic measurements of the metal microstructure [16–17]. The neutron diffraction study clearly indicates that the Iceman blade was produced by casting copper in a bivalve mold, it never underwent mechanical hardening, and it was repeatedly used in the soft state, as testified by the non invasive texture analysis of the cutting edge [17]. More extensive studies on Copper Age axes carried out by standard metallographic techniques [18] show that in many cases a recrystallized microstructure is observed, produced by heat treatment of sufficient intensity to erase the strain and deformation caused by use, perhaps followed by slight mechanical hardening of the blade and edges. Although metal hardening in the fourth millennium was technically already developed for daggers, and metal working was certainly used for ornaments, apparently the axe blades were mostly used in the soft state to favor ductility over hardness [18].

Once defined the manufacturing technique of the axe, the metal provenance is a fundamental issue to be investigated, as valuable information on metallurgical activities and metal movements in the fourth millennium BC can be retrieved. This aim is accomplished through: (*i*) the invasive microsampling of the metal for precise measurement of chemical and isotopic tracers by mass spectrometry [19], and (*ii*) the interpretation of the measured data by referring to an exhaustive geochemical dataset of known copper ores to determine the metal source [20–22].

These conditions were met early in 2016, when the scientific committee controlling the scientific sampling and investigations on the Iceman and his implements granted permission to microsample the metal inside an existing fracture [11, 15] in the talon of the axe blade (Fig 1). The fracture is likely to be the result of the repeated mechanical stress of the handle on a casting flaw present in the talon of the blade. The microsampling produced enough copper metal to perform both chemical and isotopic analyses, as requested by the scientific committee.

## Materials and methods

### Microsampling

The metal microsamples were obtained from the crack defects already existing in the talon of the axe (Fig 1). The micro-fragments were cut under an optical binocular microscope (50X) using a micro-tool equipped with a steel blade. The manual operation using a fine blade was preferred over drilling to ensure better control on the sampling position and on the amount of extracted metal, and to avoid changes in the microstructure of the fragments subsequently used for metallographic analyses. Three fragments of copper were detached, amounting to a total weight of 6.7 mg. The largest fragment was used for the measurement of the lead isotope ratios by multi-collector plasma source mass spectrometry. One smaller fragment was used for the chemical analysis by quadrupole plasma source mass spectrometry. The third micro-fragment was embedded in epoxy resin, polished, and used for metallography and electron microscopy.

### Metallography

Metallographic analysis by reflected-light optical microscopy was carried out on the cross-section of the small fragment using a Nikon Eclipse ME600 microscope operating in reflected light.

### Electron microscopy

After the metallographic analysis, the same fragment was carbon-coated and analyzed through scanning electron microscopy, both for imaging and preliminary chemical analyses by energy dispersive spectroscopy (SEM-EDS), using a Cameca CamScan MX 2500 microscope with a LaB_6_ source. The X-ray fluorescence signal was treated with SEM Quant PhiZAF software. The electron backscattered images, coupled with chemical analysis, allow better definition of the cuprite inclusions and semi-quantitative assessment of the composition of the chemical segregations.

### Quadrupole mass spectrometry

A small sample was completely dissolved in a 1:1 solution of nitric acid and hydrochloric acid and analysed by inductively coupled plasma mass spectrometry (ICP-MS). All reagents were of analytical grade and were purified by redistillation before use. The sample and calibrating solutions were prepared in milliQ Ultrapure water obtained with a Millipore Plus System. The calibration solutions were prepared by gravimetric serial dilution from multi-element standard solutions (IV-ICPMS-71A stock solution, Inorganic Ventures; IMS-103 Ultra Solutions), at nine different concentrations in the range 0.1–1000 ppb. A multi-element internal standard (Agilent technologies) was added to the sample solution in order to check for instrumental drift. The same procedure for sample preparation was used to prepare blank samples of ultrapure water and reagents.

The mass spectrometric data were measured with an Agilent Technologies 7700× ICP-MS mass spectrometer (Agilent Technologies International Japan, Ltd., Tokyo, Japan). The ICP-MS was equipped with an octupole collision cell operating in kinetic energy discrimination mode. It was used for the removal of polyatomic and argon-based interferences. The instrument was optimized to achieve optimum sensitivity and stability according to the manufacturer's recommendations. Detection limits are calculated following the reported procedures [23]. An attempt was also made to detect organic contaminants from the sample, using previously reported procedures [24], but none was observed above background level.

### Multi-collector mass spectrometry

To investigate the lead isotope ratios the third aliquot of metal extracted from the axe (5.4 mg) was dissolved in hot triply distilled concentrated nitric acid by high-pressure microwave digestion in sealed PTFE vessels. As described in Villa [25], the dissolved lead was purified using the SrSpec™ resin (EIChroM Industries). About 100ml of resin are filled in a 3-mm diameter hand-made PTFE column. The height to width ratio is approximately 4. The sample solution is loaded in 0.5 ml 1M HNO_3_, 1.5 ml of which is also used to wash out the matrix metals, while Pb is very strongly retained on the resin. Pb is then eluted with 3 ml 0.01 M HNO_3_ and is ready for analysis.

The measurement of lead isotope ratios were performed using a Thermo Scientific Neptune Multi-collector ICP–MS instrument at the Institut für Geologie, University of Bern (Switzerland). This instrument is equipped with a double-focusing geometry. The Faraday collector array allows the simultaneous acquisition of masses 202 to 209. The sample introduction system consisted of an auto-aspirating low-flow (50 ml min^–1^) Apex desolvating nebulizer (ESI Scientific, Omaha, NE, USA) mounted on to a combined cyclonic/double-pass spray chamber made of quartz glass. Potential isobaric interference of ^204^Hg on ^204^Pb was controlled and, if necessary, corrected for by monitoring the ^202^Hg signal. Hydride formation (PbH) was monitored on mass 209 and never detected. Mass fractionation was monitored by adding a small quantity of Tl, which has a known ^203^Tl/^205^Tl ratio, fractionated by the same mechanism as Pb and does not interfere with Pb isotope measurements [26]. The measurement accuracy was controlled with frequent measurements of the NIST SRM 981 standard reference material interspersed with the sample measurements. The measured isotopic composition for SRM 981 was indistinguishable from the certified value and the recent, more precise literature measurements [27], so that no adjustment of the measured ratios was necessary. The external reproducibility on the SRM 981 reference material over the measuring period of several days amounted to ±0.015% (2s).

Ötzi’s axe is preserved intact at the South Tyrol Museum of Archaeology in Bolzano (Italy). All necessary permits were obtained for the described study, which complied all the relevant regulations. Microsampling and analyses permits for the project “Analisi dei rapporti isotopici del Pb sul rame metallico dell’ascia dell’Uomo venuto dal ghiaccio” were provided in written form dated March 31^st^, 2016 by the Director of the “Azienda Musei Provinciali” and the Director of the “Museo Archeologico dell’Alto Adige”, after the positive response of the Scientific Council of the Museum.

## Discussion

The results of the chemical analysis of the impurities contained in the copper metal performed by mass spectrometry (Table 1) agree with the early determinations performed by surface X-ray fluorescence spectrometry [14–15]. Arsenic and silver at a concentration of about 0.4 wt% and 0.1 wt%, respectively, are the only elements that are clearly detectable by X-ray fluorescence spectrometry, as all the others are around or below the limit of detection of the technique.

**Table 1:** Results of the elemental and isotopic analyses performed on the metal of the Iceman copper axe.

*Element*	*ppm*	*e*.*s*.*d*.
As	4360	30
Ag	956	7
Fe	204	1
Ni	202	1
Bi	177	1
Se	66	1
Zn	29.8	0.2
Pb	23.0	0.2
Sb	18.6	0.1
Te	6.8	0.5
Au	5.08	0.04
Sn	5.45	0.04
V,Cr,Mn,Co,Pd,Pt	<1	
***isotopic ratio***		
^206^Pb/^204^Pb	18.774	0.016
^207^Pb/^204^Pb	15.706	0.013
^208^Pb/^204^Pb	39.029	0.032

**Fig 2:**
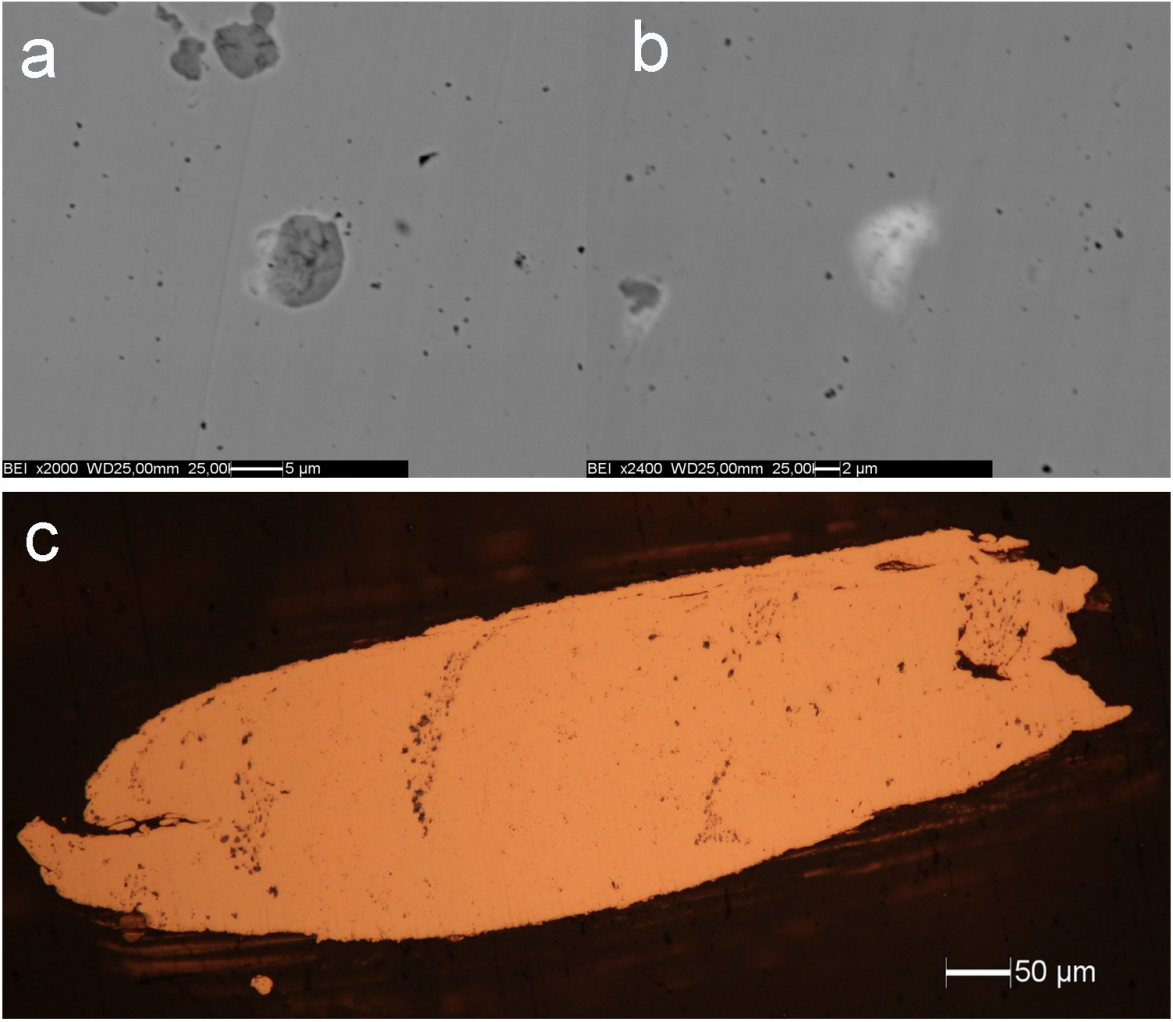
Micro-inclusions present in the copper matrix. (a) Backscattered electron images of the cuprite inclusion (dark grey) surrounded by a lighter rim rich in As (30.7%) and Bi (20.3%); (b) white segregation rich in As (11.8%),Se (9.5%),Bi (4.2%), and Pb (13.2%). Both inclusions are about 5 μm across; (c) reflected-light optical micrograph. The total length of the fragment is 870 μm.

High sensitivity plasma-source quadrupole mass spectrometry allows reliable detection of additional elements such as Fe, Ni, and Bi at sub-ppm concentrations. Backscattered electron images (Fig 2A and 2B), coupled with point EDS analyses, clearly show that As (in the range 4.5–42.3%), with minor concentrations of Bi (3.6–20.27%) and Se (observed only in two occurrences, ~9.5%), forms micro-segregations in the copper matrix, whereas Ag is dispersed in the copper metal. In fact, As, Bi and Se were detected at relatively high concentrations in micro-segregations indicating that they are enriched in the segregations. Moreover, they were undetected in the surrounding matrix by EDS analysis and measured at low concentrations by ICP-MS in the bulk,. Sulphur was not detected by EDS analysis in any of the point analyses nor by mass spectrometry in the bulk. Swarms of cuprite inclusions (Cu_2_O) are readily visible in the reflected-light image (Fig 2C). Cuprite inclusions are thought to result from the nearly complete immiscibility of oxygen in copper, both in liquid and solid states. Cu_2_O can originate from reactions occurring when copper is molten, since copper absorbs both oxygen from the air and hydrogen from the decomposition of water vapour. When the copper cools and crystallizes the hydrogen is expelled and cuprite inclusions occur in the matrix. Consistently, the presence of cuprite inclusions and the absence of sulphur indicate the use of sulphide-poor copper mineralizations or highly oxidizing conditions of casting. The alignment of the swarms of cuprite inclusions are typical of the eutectic microstructures forming during cooling and are unrelated to the deformations induced during the cutting operations.

The chemical analysis showed a Pb concentration sufficient for the measurement of the lead isotope ratios by multi-collector mass spectrometry. The measured ratios are reported in Table 1. The lead isotope data measured on the Iceman axe microsample were interpreted by the use of an extensive dataset encompassing most of the known Alpine copper deposits that were sampled and measured in the frame of the ongoing Alpine Archaeocopper Project [22, 28–29]. The dataset also contains the critically revised lead isotope data available in the literature [30–32 and the literature cited within these works], including the Iberian peninsula, Tuscany and the Italian Apennines, Sardinia, Central Europe from France to Slovakia, the Balkans from Romania to Greece, Anatolia (including Taurus and Pontic mountains), and the Aegean (including Cyprus and Crete). Ore data from Scandinavia, the British Isles, North Africa, and the Levant are also present in the dataset and they were preliminary tested, but were not used in the final data analysis because of the poor match with the measured lead isotope ratios, and because these regions are considered very unlikely sources for the metal objects found in the Alps during the Copper Age.

Recent analysis based on kernel density estimation [33] and calculation of point-to-point Euclidean distances [34] in the 3D space of the measured lead isotope data (i.e. ^206^Pb/^204^Pb vs ^207^Pb/^204^Pb vs ^208^Pb/^204^Pb) shows that it is indeed possible to limit the number of conceivable copper sources and, in many cases, reliably assess the provenance of the metal [22, 35]. When ambiguities persist because of overlapping lead isotopic signatures of different ore deposits, it is frequently possible to resolve the ambiguities on the basis of the chemical composition of the metal, by using the trace element signature [19, 35–37].

**Fig 3:**
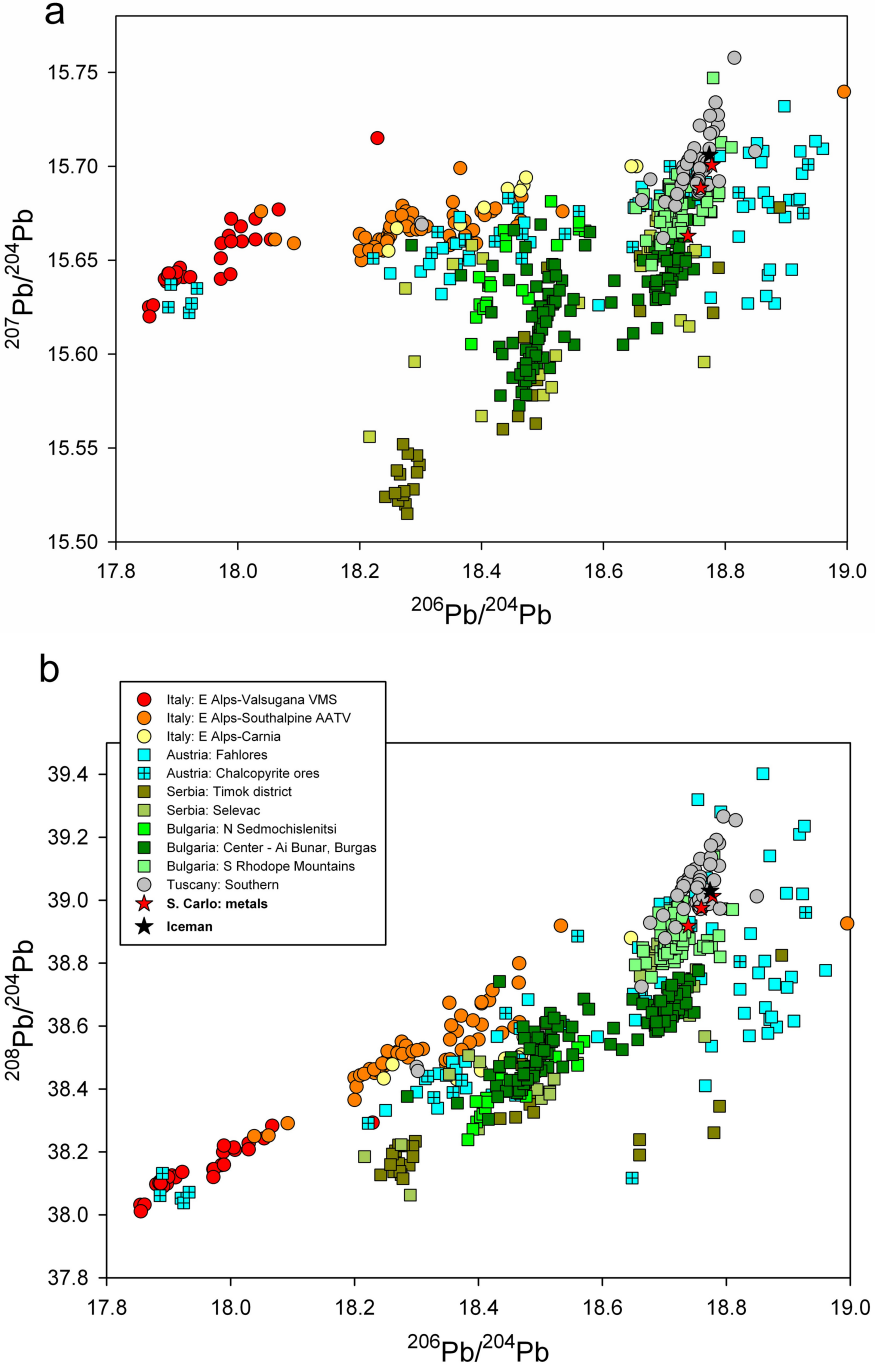
2D projections of the measured lead isotope ratios, compared with the available data from the literature [22, 28–32 and literature cited therein]. The two most widely used bivariate plots are shown: (a) ^206^Pb/^204^Pb vs ^207^Pb/^204^Pb plot; (b) ^206^Pb/^204^Pb vs ^208^Pb/^204^Pb plot.

Fig 3 shows the measured Pb isotope ratios for the metal of the Iceman axe (black star) compared with the most likely copper deposits following the ranking of the closest calculated Euclidean distances. The ranking list shows that out of the twenty closest points in the dataset half of them (and all five closest points) belongs to a very well defined region: the mines of Southern Tuscany located around Campiglia Marittima and the Colline Metallifere. Luckily, the Tuscan ores are very well discriminated against most of the deposits present in the dataset. Concerning the available data for the Eastern Alpine region, all the ore fields of the Italian Southern Alpine domain (Trentino, Alto Adige, Veneto [22], that is the red, orange and yellow circles, respectively, in Fig 3) have markedly lower ^206^Pb/^204^Pb values. Several of the chalcopyrite-based mines in Austria (Styria, Carinthia, Salzburg, marked with the crossed blue squares) also have the same isotopic character and can be safely excluded. The few crossed blue squares plotted on the right side of the Tuscan ores, at higher ^206^Pb/^204^Pb, belong to the chalcopyrite-based Mitterberg ore field [38], which however has a marked radiogenic character and mostly plots way out of the selected isotopic range, at substantially higher ^206^Pb/^204^Pb and ^207^Pb/^204^Pb values. The Austrian mines, isotopically overlapping with the Tuscan field (mainly from the Montafon and the Inn Valley in Tyrol, marked with open blue squares), are all related to fahlerz-based ores (*i*.*e*. mineral assemblages based on tetrahedrite-tennantite minerals) and contain substantial quantities of Sb, which is negligibly present in the copper axe (Table 1). The early chemical analyses already showed that the Iceman axe is incompatible with the Tyrolean deposits because of the lack of measurable antimony [15].

**Fig 4:**
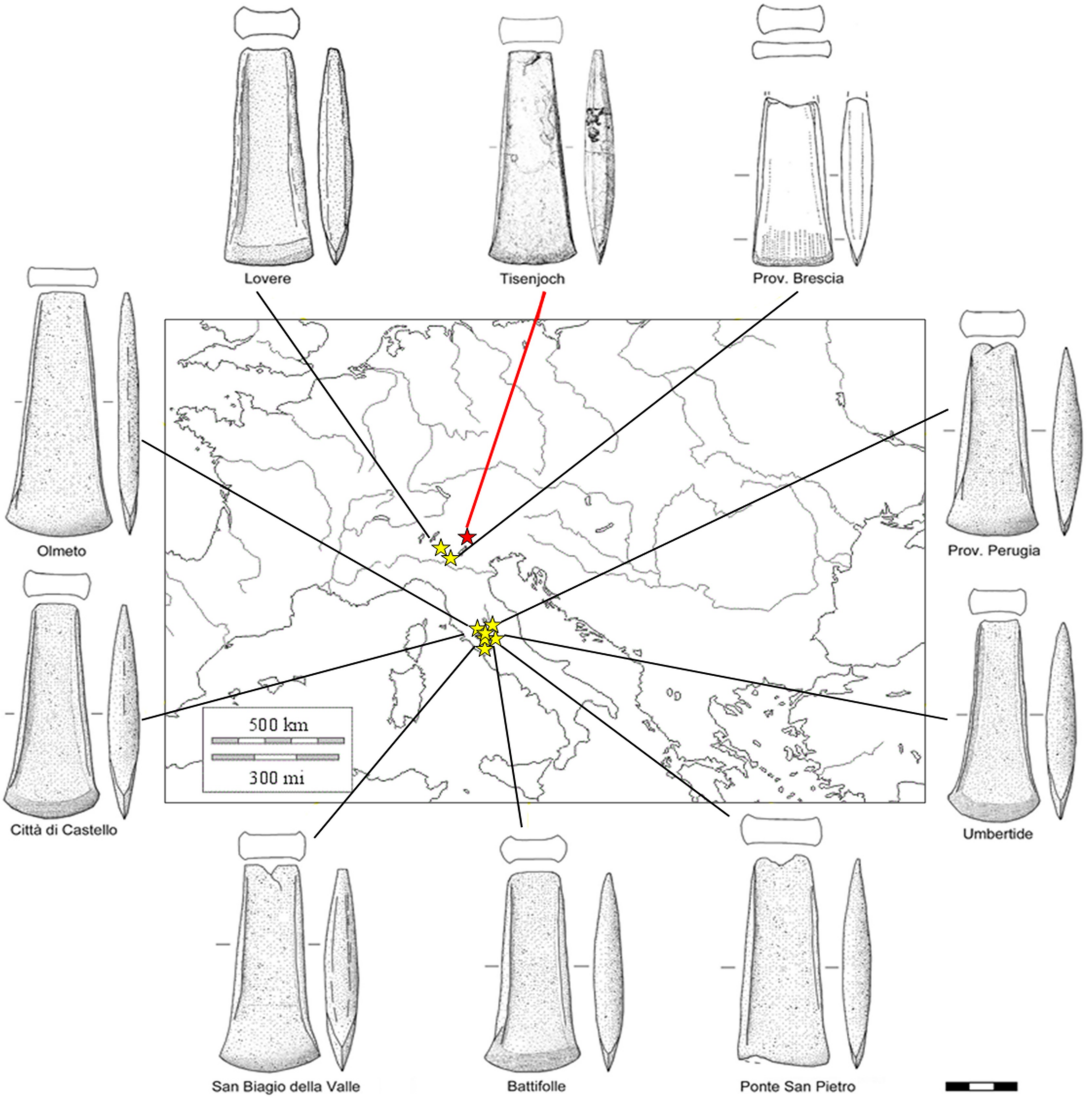
Typological comparison between Copper Age flanged axes. Tisenjoch = Iceman axe (BZ), Lovere (BG), Brescia (BS), Olmeto (PG), Perugia (PG), Città di Castello (PG), Umbertide (PG), San Biagio della Valle (PG), Battifolle (AR), and Ponte San Pietro (VT). Scale: 1:3.

It can be therefore safely inferred that the copper metal composing the axe was extracted from Southern Tuscan ores and not from Alpine or Balkanic ores, despite the fact that copper deposits in both areas were certainly known and exploited during the Copper Age [39–45]. This apparently unexpected result is actually in line with the recent re-evaluation of the prehistoric metallurgical activities in the Tuscan area. The recent investigations of the archaeological evidence at the Copper Age site of San Carlo, near S. Vincenzo, Livorno, prove that it was a center of substantial metallurgical activity (copper reduction and refining) at about the same time as that of the Iceman [46]. Radiocarbon dating of charcoal fragments present within the metallurgical slags of San Carlo indicates a calibrated age around 3400–3100 BC (95% confidence level at 2σ), which is perfectly compatible with the assessed age of the Iceman, estimated at 3350–3000 BC (average of three radiocarbon dates on the axe haft, 95% confidence level at 2σ) [11]. Furthermore, the chemical composition of some of the metal drops found at San Carlo is very similar to the one observed in the Iceman axe: they consist of very pure copper with segregations of As and Bi [46]. The lead isotope signatures are also consistent with local ores. Of course it is not possible to prove a direct production of the axe in the San Carlo area, nor is it possible to discern whether the raw metal or rather the object itself originated in the area. It is unlikely that in the late 4th millennium BC San Carlo was the only active metallurgical site. However the close chemical and isotopic link between the Iceman axe and Southern Tuscany is hard to dismiss. Furthermore, reassessment of the typological similitude of the Iceman axe to other coeval axes found in North-Central Italy (Lovere [47], Brescia [48–49], Olmeto [48], Perugia [48], Città di Castello [47], Umbertide [47], San Biagio della Valle [47, 50], Battifolle [48], and Ponte San Pietro [51]) indicates that indeed the “weakly flanged axe”-type family was rather common in the area (Fig 4).

The presence of active copper metallurgy in Central Italy during the second half of the fourth millennium BC and the Tuscan origin of the Iceman copper seem to support the recent re-evaluation of the whole metallurgy related to the so-called Rinaldone culture. It was diffused in the region and in the past traditionally attributed to the Bell Beaker event (second half of the third millennium BC). However, recent reassessment of the archaeological evidence based on radiocarbon measurements indicates the backdating of the Rinaldone culture to the fourth millennium BC [52–53]. In view of these results, it will be very interesting in the future to evaluate the provenance and diffusion of the metals related to the northern Italian cultures of Spilamberto (South of the Po River), Remedello (North of the Po River) and Tamins-Carrasso-Isera (Central Alps). These populations may have been related to the northbound trade of Tuscan copper.

**Fig 5:**
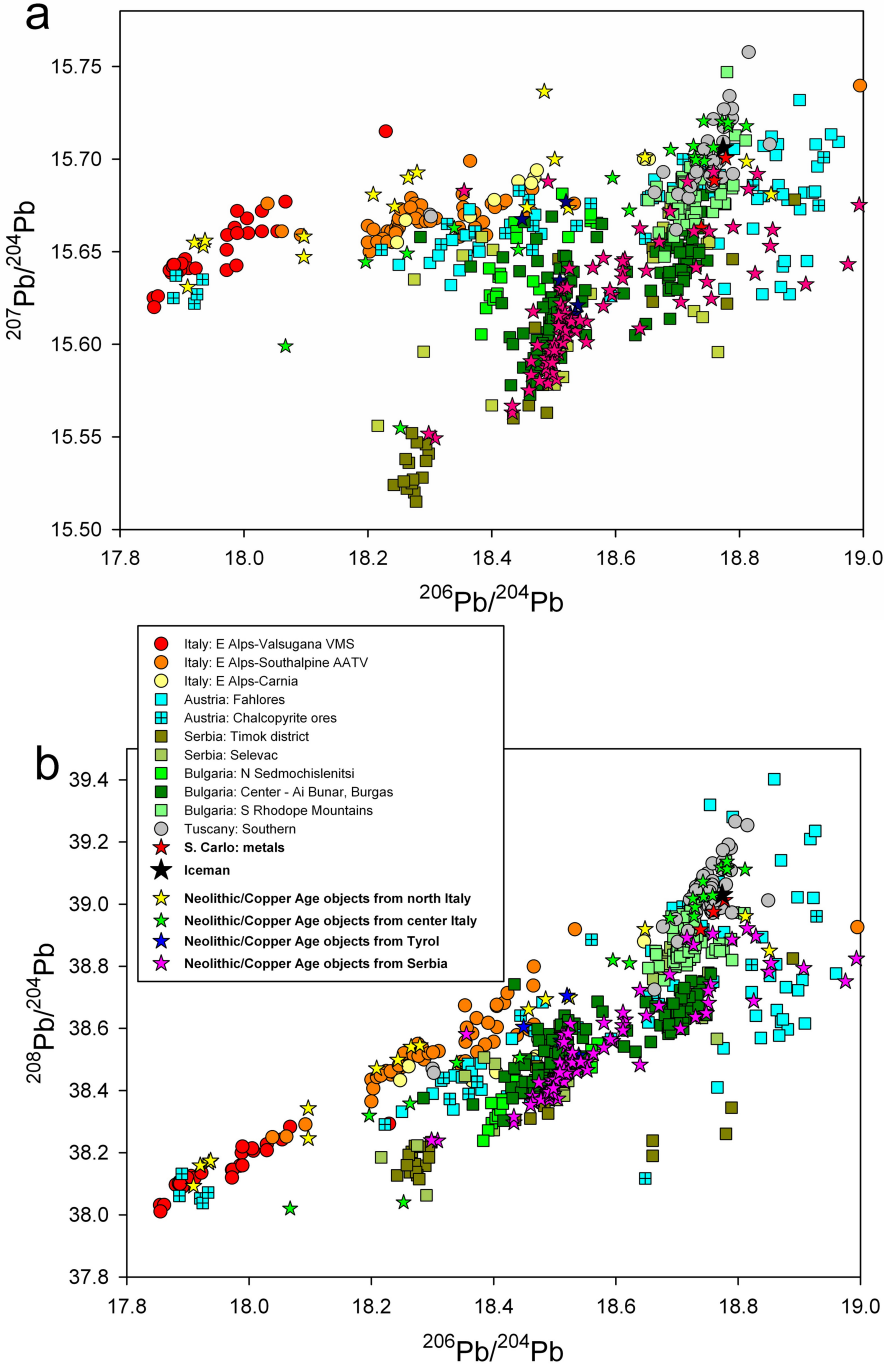
2D projections of the available lead isotope data for Neolithic and Copper Age objects from Italy [22, 31, 39, 54–55], Tyrol [41], and Serbia [42–56] (from the late 5^th^ millennium to the early 3^rd^ millennium BC). (a) ^206^Pb/^204^Pb vs ^207^Pb/^204^Pb plot; (b) ^206^Pb/^204^Pb vs ^208^Pb/^204^Pb plot.

In Fig 5 we tentatively plotted all published isotopic data for metal objects broadly dated to the fourth and third millennia BC, which were found north and south of the Alpine region. Apart from a few of objects that were obviously traded on long distances and that will not be discussed here, there is an evident and surprisingly clear pattern of provenance and diffusion of copper metal of the objects. Virtually all of the objects found south of the Alps (northern and central Italy) were made of Tuscan or southern Alpine copper [22, 31, 39, 54–55], whereas all of the objects found north (Tyrol) [41] and east (Serbia) [42–56] of the Alps were produced from Balkanic copper, mainly from Serbia and Bulgaria. The Austrian copper sources in Tyrol and Salzburg (such as Schwaz and Mitterberg) evidently came to be exploited at later times. The observed patterns indicate that the Alps in the early Copper Age were more a barrier than a link, at least as far as the metal trades are concerned [57]. These results may indeed induce a revision of the long held assumptions about the early diffusion of Balkanic copper into Italy: the Iceman axe blade is indeed shining of a new light.
