# Correction: Long-distance connections in the Copper Age: New evidence from the Alpine Iceman's copper axe

**DOI:** 10.1371/journal.pone.0189561

**Published:** 2017-12-07

**Authors:** Gilberto Artioli, Ivana Angelini, Günther Kaufmann, Caterina Canovaro, Gregorio Dal Sasso, Igor Maria Villa

The first sentence of the sixth paragraph in the Discussion section should have cited references 39–45, 56 instead of 39–45. The correct sentence should read: It can be therefore safely inferred that the copper metal composing the axe was extracted from Southern Tuscan ores and not from Alpine or Balkanic ores, despite the fact that copper deposits in both areas were certainly known and exploited during the Copper Age [39–45, 56].

The third sentence of the eighth paragraph in the Discussion section should have cited reference 42 instead of references 42–56. The correct sentence should read: Virtually all of the objects found south of the Alps (northern and central Italy) were made of Tuscan or southern Alpine copper [22, 31, 39, 54–55], whereas all of the objects found north (Tyrol) [41] and east (Serbia) [42] of the Alps were produced from Balkanic copper, mainly from Serbia and Bulgaria.

The Fig 5 legend should have cited reference 42 instead of 42–56. The correct Fig 5 legend is: **Fig 5. 2D projections of the available lead isotope data for Neolithic and Copper Age objects from Italy [22, 31, 39, 54–55], Tyrol [41], and Serbia [42] (from the late 5**^**th**^ **millennium to the early 3**^**rd**^ **millennium BC).** (a) ^206^Pb/^204^Pb vs ^207^Pb/^204^Pb plot; (b) ^206^Pb/^204^Pb vs ^208^Pb/^204^Pb plot.
